# Pediatric Kienböck’s Disease in an 11-Year-Old Boy: A Case Report

**DOI:** 10.7759/cureus.47788

**Published:** 2023-10-27

**Authors:** Ahmad Al-Zoubi, Fadi AlRousan, Saab Mestarihi, Mohamad Olaimat, Ehab Azzam, Ahmad Almigdad

**Affiliations:** 1 Department of Orthopedics, Royal Medical Services, Amman, JOR

**Keywords:** avascular necrosis of lunate, lunatomalacia, lunate bone, pediatric, kienböck’s disease

## Abstract

Kienböck’s disease is a rare condition in the pediatric age group, with little agreement on its natural history and the best treatment option. Typically, these patients present with vague wrist pain and a variable degree of joint motion restriction. Diagnosis is mainly based on clinical suspicion and MRI findings, as radiographs do not show significant changes in the early stages of the disease. Prognosis is better in children than in adults due to the good healing capacity in this age group.

This study will report on an 11-year-old boy diagnosed with Kienböck's disease who underwent surgical treatment to temporarily offload the diseased lunate. Clinical and radiographic recovery was observed at the six-month follow-up.

## Introduction

Kienböck’s disease is an idiopathic osteonecrosis of the lunate bone, with subsequent stages of fragmentation and collapse that lead to progressive pain and decreased wrist range of motion. The disease is well-described in adults and mainly affects the dominant hand of male patients between the ages of 20 and 40 years [1]. Different treatment strategies have been investigated based on the disease stage described by Lichtman et al. [2]. The treatment aims to preserve the shape of the lunate bone and prevent wrist osteoarthritic changes.

Kienböck’s disease is extremely rare in the pediatric age group, with few cases reported in the literature [3]. This report presents an 11-year-old boy diagnosed with stage two Kienböck’s disease, based on MRI, who underwent surgical treatment with scaphoid-trapezoid-trapezoidal (STT) wiring for six weeks. Clinical and radiographic recovery were observed at six months of follow-up.

## Case presentation

**Figure 1:**
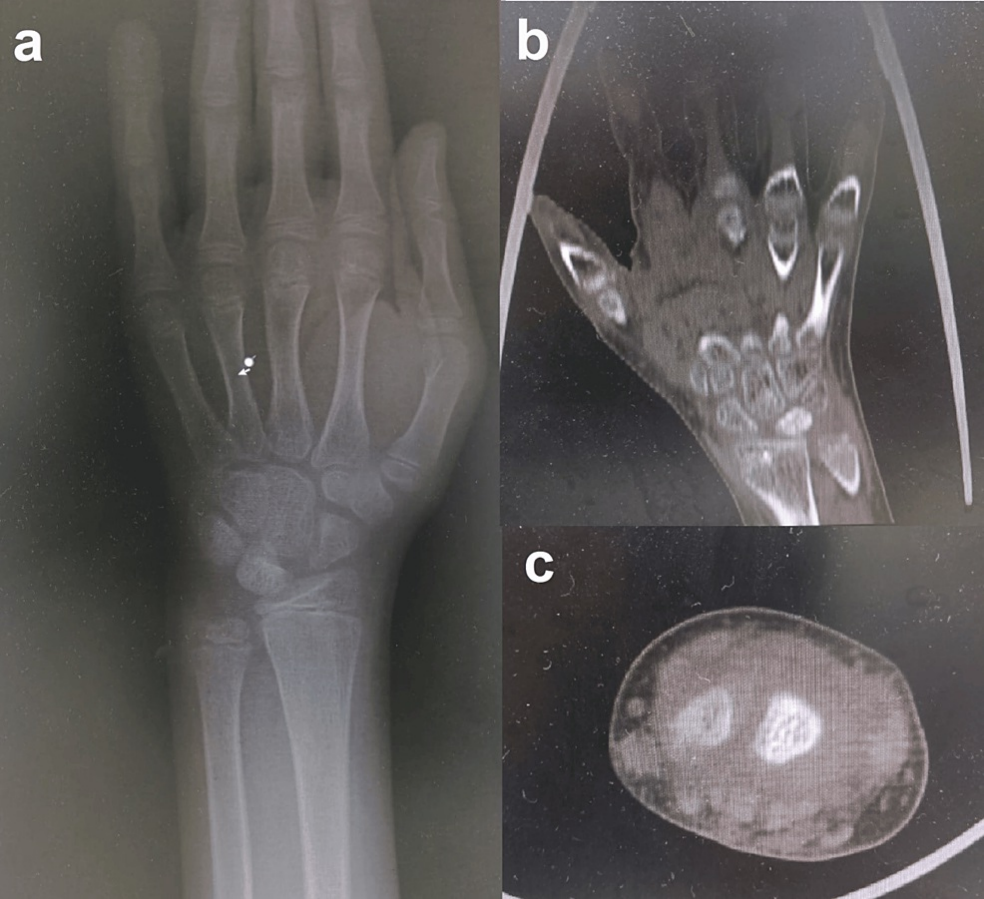
(a) Posterior-anterior left wrist radiographs demonstrated lunate bone sclerosis and widening of scapholunate space. (b, c) Coronal and axial wrist CT scans showed lunate bone sclerosis.

**Figure 2:**
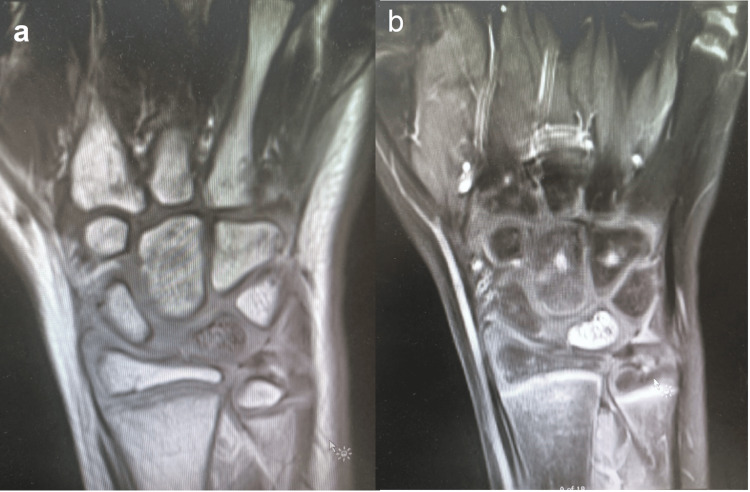
The altered bone marrow signal of lunate bone, which appears hypointense on T1-weighted image (a) and hyperintense on short tau inversion recovery (STIR) (b), represents a bone marrow edema but an intact cortex with no loss of volume.

An 11-year-old male presented with left wrist pain of two months’ duration. The child did not recall a history of trauma; the pain was described as vague pain unrelated to activity. Examination revealed localized tenderness on the dorsum of the wrist and reduced wrist flexion and extension to 55° and 30°, respectively, compared to 90° and 90° in the contralateral wrist. The initial radiograph showed sclerosis of the lunate bone and widening of the scapholunate space, yet, it was comparable to that of the contralateral wrist. CT scan demonstrated sclerosing of the lunate bone (Figure 1). MRI showed abnormal signal density in the lunate bone in all consequences, hypointense signal density on T1-weighted images, and hyperintense signal in short tau inversion recovery (STIR) MRI, which is consistent with avascular necrosis of the lunate bone (Figure 2).

**Figure 3:**
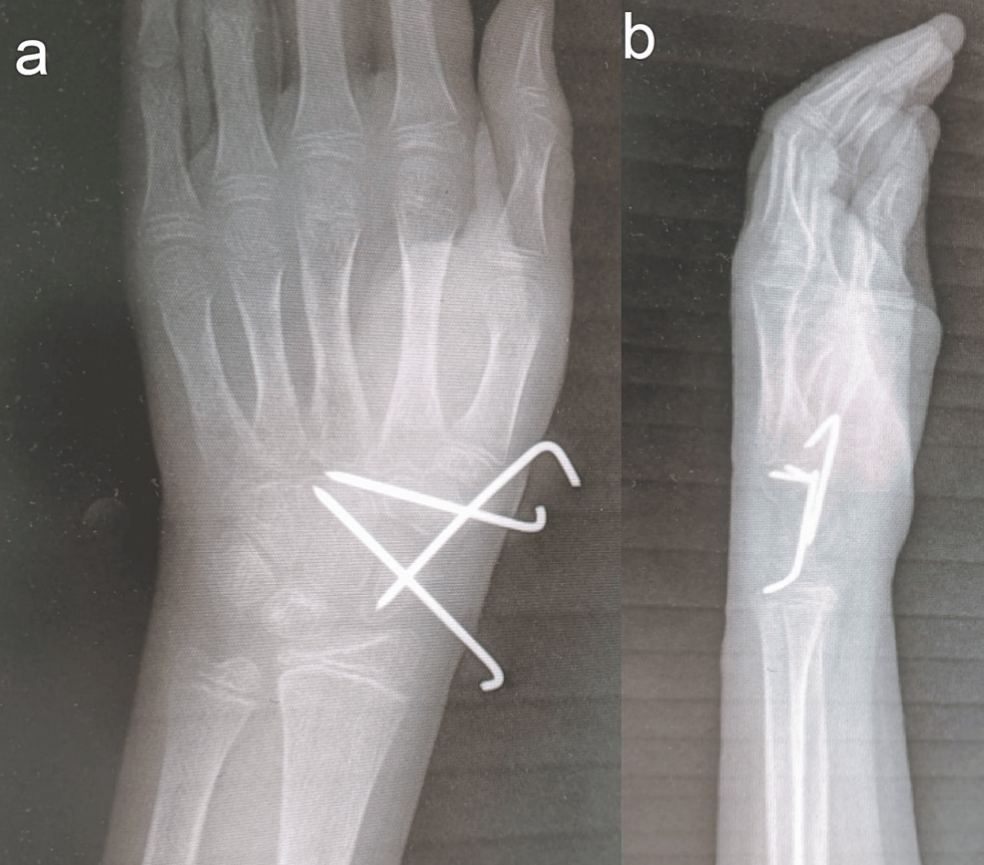
(a) Posterior-anterior and (b) lateral wrist radiographs showed scaphoid-trapezoid-trapezoidal wiring.

Therefore, the child underwent surgery to offload the lunate bone by STT wiring. The surgery was performed under general anesthesia and a fluoroscope. Three 1.8 mm Kirschner wires were inserted percutaneously; the first pin was inserted from the trapezium to the trapezoid and capitate, the second pin from the scaphoid to the capitate, and the third pin from the trapezium to the scaphoid (Figure 3). A short arm cast was applied for six weeks.

**Figure 4:**
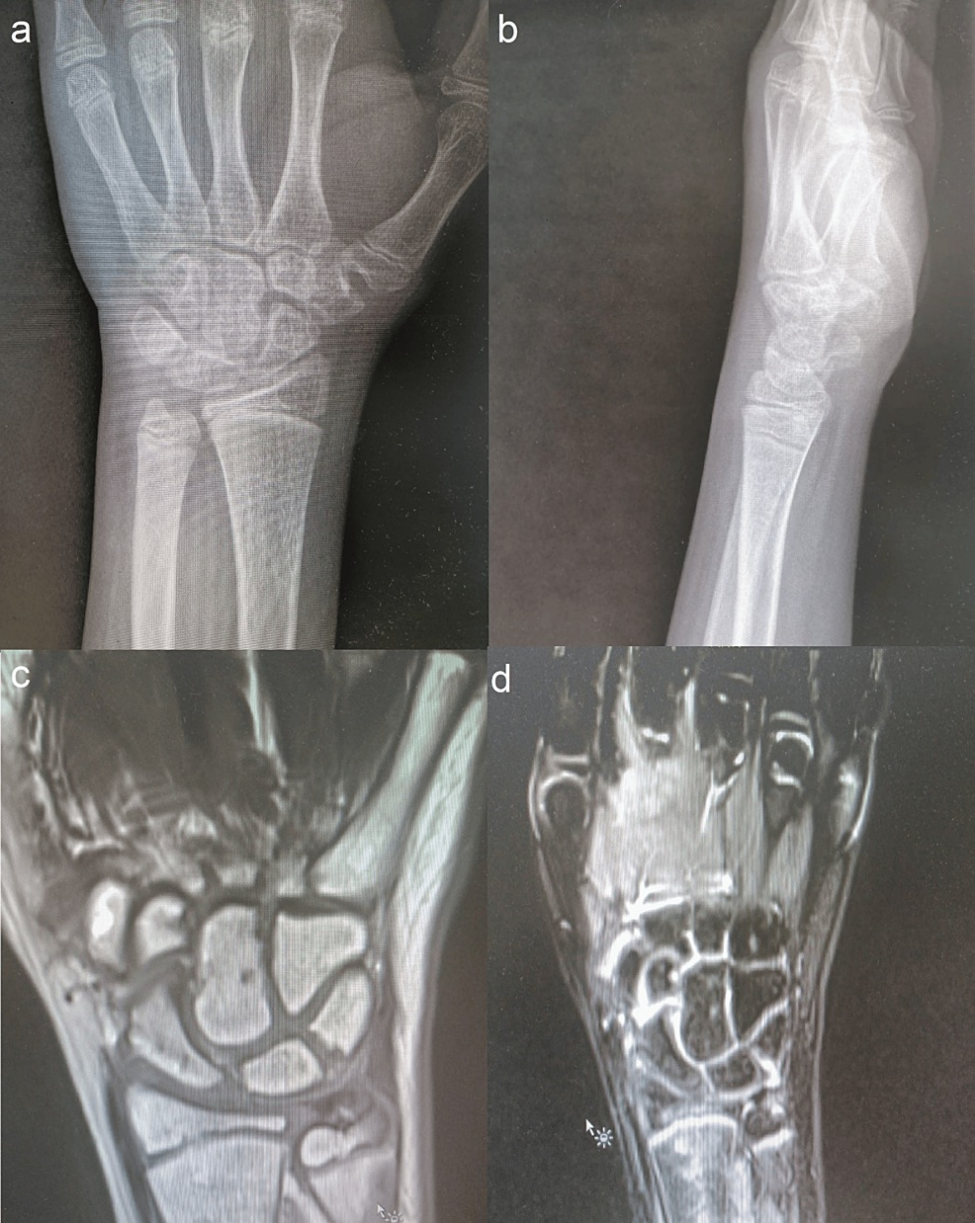
(a, b) Posterior-anterior and lateral left wrist postoperative radiographs at six months showed restoration of bone density. (c, d) Coronal T1 and T2 left wrist MRI showed signs of revascularization, including the return to normal intensity.

**Figure 5:**
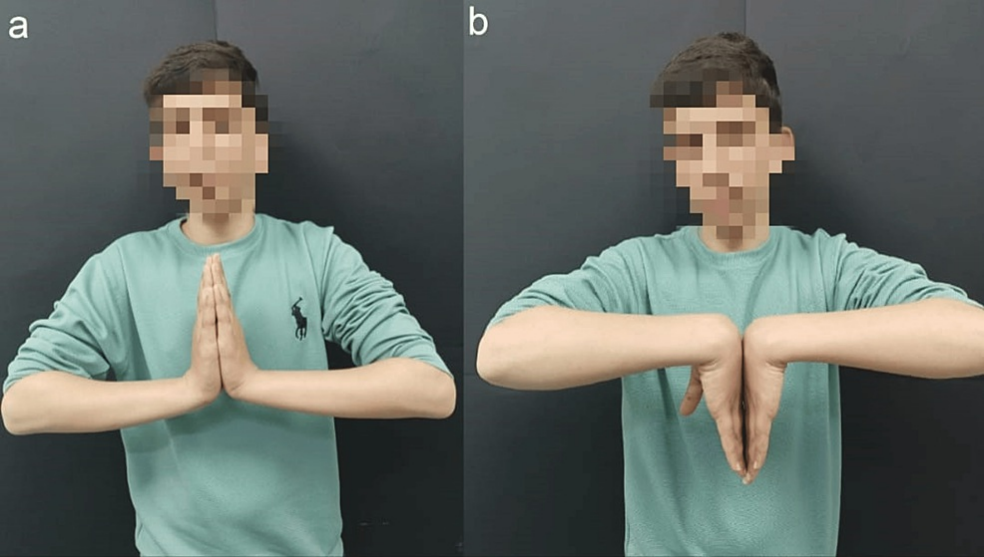
Postoperative wrist range of movement at six months demonstrated restoration of wrist extension (a) and flexion (b) to 90°.

Kirschner wires were removed at six weeks, and a removable wrist splint was applied for a further four weeks. The child reported resolution of pain at four months. Radiographs at six months showed a return to normal bony density, and MRI revealed the resolution of previous MRI changes and maintained lunate bone shape (Figure 4). The child regained a range of movement comparable to the contralateral side (Figure 5).

## Discussion

Kienböck’s disease, also known as lunatomalacia, is an idiopathic osteonecrosis of the lunate bone. Usually present in males in the middle age group. It mainly involves the dominant hand. Pain, decreased wrist range of motion, and weak grip are common clinical presentations.

Although the etiology of the disease is not well understood [4], many factors predispose to the disease, such as repetitive trauma, negative ulnar variance, and variability in vascular morphology. Initial plain radiographs are normal, and MRI is the modality of choice for diagnosing early disease and ruling out ulnar impaction. Lichtman classified Kienböck’s disease based on imaging changes on radiographs and MRI guide treatment options according to the stage.

Pediatric Kienböck’s disease is extremely rare, and there is little evidence to guide optimal treatment in this age group [5]. The natural history of this disease is unknown, and there are no available data to compare different treatment modalities. However, there is an excellent capability of healing and remodeling in pediatric patients, with a favorable outcome expected in both surgical and non-surgical [6] treatment modalities in terms of clinical and radiographic recovery, despite some observed residual loss of height at the radial aspect of the lunate bone [7]. Kalb et al. reported on three pediatric patients with Kienböck's disease, one was 17 years old and exhibited disease progression despite temporary scaphotrapezoidal joint transfixation. The authors posit that pediatric and juvenile patients, particularly those under 14 years old, have the potential for spontaneous remodeling and revascularization, leading to favorable outcomes [3]. Some data suggest that children older than 12 years might have disease progression similar to adults [8].

Non-surgical treatment with casting and rest is recommended in the early stage of the disease. However, different surgical treatments have been described in more advanced cases, such as radial shortening [9] and temporary STT wiring [10], which are used to treat advanced cases of Kienböck’s disease.

Good functional recovery has been observed in pediatric patients with all described treatment modalities, with no superiority of any of these treatment methods. If Kienböck’s disease is compared with other idiopathic avascular disorders in the pediatric population, like Kohler’s disease or Hegemann’s disease, many patients do well with non-surgical treatment.

## Conclusions

Our case study highlights the importance of considering Kienböck’s disease in the differential diagnosis of wrist pain in the pediatric age group. Further studies are needed to determine the best treatment option for this rare disease in pediatrics.
